# Streamlining care through patient navigation: a retrospective cohort study of timely anti-HER2 therapy in early breast cancer in a low-middle income country

**DOI:** 10.1186/s12913-025-13606-8

**Published:** 2025-11-08

**Authors:** Emad Shash, Fatema Alaa, Engy Maher, Julia F. Rostom, Alaa Ibrahim, Rania Said, Nada Abou El-Kheir, Mona Elhosary, Reem Eid

**Affiliations:** 1 Medical Oncology Department, National Cancer Institute, Cairo University, Fom El Khalig Square, Cairo, Egypt; 2 Breast Cancer Comprehensive Center, National Cancer Institute, Cairo University, Cairo, Egypt; 3 Patient Navigation Unit, Breast Cancer Comprehensive Center, National Cancer Institute, Cairo University, Cairo, Egypt; 4 Cancer Epidemiology & Biostatistics Department, National Cancer Institute, Cairo University, Cairo, Egypt

**Keywords:** Breast cancer, HER2-positive, Dual HER2 blockade, Patient navigation, Treatment delay, Low- and middle-income country (LMIC)

## Abstract

**Background:**

Timely initiation of therapy is critical for patients with HER2-positive early breast cancer, especially in low- and middle-income countries (LMICs) where health-system constraints delay care. We evaluated whether a Patient Navigation Program could reduce time from registration to initiation of dual anti-HER2 therapy in Egypt.

**Methods:**

Retrospective cohort study at the Breast Cancer Comprehensive Center (BCCC). Trained navigators tracked diagnostics, scheduled multidisciplinary tumor board (MDT), prepared/submitted Ministry of Health (MOH) approval files, monitored approval, and booked the earliest infusion slot. The primary endpoint was time from registration to therapy start; secondary endpoints were prespecified intervals—T1 (registration→MDT), T2 (MDT→MOH submission), T3 (MOH submission→MOH approval), and T4 (MOH approval→therapy start), time to surgery, and pathological complete response (pCR). The primary analysis compared symmetric six-month windows: July–December 2022 (without navigation) vs. January–June 2023 (with navigation). A sensitivity analysis included all eligible patients: May–December 2022 vs. January–December 2023. Two-sided *p* < 0.05 was significant.

**Results:**

In the primary analysis, navigation significantly shortened MOH approval → therapy start (T4) (*p* = 0.008), while T1–T3 and total time showed non-significant differences (total: *p* = 0.127). pCR was similar (78/115 [67.8%] vs. 81/117 [69.2%], *p* = 0.818). In the sensitivity analysis (*N* = 441), total time decreased from 146.2 ± 76.6 to 121.6 ± 50.4 days (–24.6 days, *p* < 0.001), driven by a large improvement in T4 (32.4→20.8 days; − 11.6 days, *p* < 0.001) while pCR remained comparable, although not statistically significant (64.1% vs. 69.9%, *p* = 0.76).

**Conclusion:**

In an LMIC tertiary center, a Patient Navigation Program significantly accelerated the post-approval step to treatment and, across the full year, shortened the overall time from registration to initiation of dual anti-HER2 therapy despite rising volumes. These system-level gains, support navigation as a scalable, equity-promoting strategy aligned with World Health Organization (WHO)Global Breast Cancer Initiative priorities; prospective multicenter evaluations incorporating patient-reported outcomes and cost-effectiveness are warranted.

**Supplementary Information:**

The online version contains supplementary material available at 10.1186/s12913-025-13606-8.

## Background

Breast cancer is the most diagnosed cancer and the leading cause of cancer death among women globally [1]. In 2020, an estimated 2.3 million new cases and 685,000 deaths were reported, with a disproportionate burden in low- and middle-income countries (LMICs) where constrained systems drive later diagnosis and treatment delays [1, 2]. Timely therapy is critical: longer intervals to treatment are consistently associated with more advanced disease at presentation and poorer survival [3–5].

Human epidermal growth factor receptor 2 (HER2)–positive breast cancer is biologically aggressive but highly curable when modern therapy is delivered without delay. In the neoadjuvant setting, dual HER2 blockade (trastuzumab plus pertuzumab) with chemotherapy significantly increases pathological complete response (pCR) versus chemotherapy alone or single-agent HER2 targeting [6]. Because pCR correlates with lower recurrence and improved survival, delays in initiating HER2-targeted treatment may compromise the probability of cure.

At the Breast Cancer Comprehensive Center (BCCC), National Cancer Institute, Cairo University (Cairo, Egypt), the standard pathway for HER2-positive early breast cancer includes neoadjuvant chemotherapy plus dual anti-HER2 therapy (trastuzumab + pertuzumab), definitive surgery, and adjuvant systemic therapy and/or radiotherapy as indicated. Within the public sector, starting dual anti-HER2 therapy requires Ministry of Health (MOH) approval based on a complete dossier (histopathology/receptor status and baseline assessments). This administrative step, alongside communication gaps and patient-level barriers (navigating appointments, document acquisition, health literacy), can introduce clinically meaningful delays—challenges echoed in other Egyptian public cancer centers. Egypt’s Presidential Initiative for Women’s Health (launched 2019) has expanded screening and access; by March 2022, >16.5 million women had been screened, and the government committed to providing innovative cancer therapies free of charge to eligible patients [7]. Realizing these gains, however, depends on parallel improvements in care coordination after diagnosis.

Patient navigation is an established strategy to reduce delays and inequities. Originating in 1990 at Harlem Hospital, navigation for underserved patients substantially improved breast cancer outcomes (five-year survival ~ 70% with-navigation versus 39% without) [8]. Across diverse settings, navigators—nurses, social workers, or trained laypersons—coordinate appointments, facilitate communication, and resolve logistical barriers, shortening time from abnormality to diagnosis and from diagnosis to treatment, particularly for vulnerable groups [9–15]. International bodies, including the Breast Health Global Initiative (BHGI) and WHO, endorse navigation as part of comprehensive cancer control in LMICs [15].

Aligned with these directives, the Breast Cancer Comprehensive Center (BCCC) established a dedicated Patient Navigation Program in 2022, the first of its kind in our region [16]. The program proactively guides patients from first visit through treatment completion, targeting expedited multidisciplinary team review, MOH approval, and therapy scheduling, while reinforcing patient education. This study evaluates the program’s impact on timeliness of dual anti-HER2 therapy initiation and downstream outcomes (including pCR), with the hypothesis that navigation meaningfully shortens the interval from registration to treatment start and advances alignment with international benchmarks.

## Methods

### Intervention: patient navigation program

**Pre-implementation (without navigation).** There was no standardized, centralized process to coordinate diagnostics, multidisciplinary tumor board (MDT) scheduling, Ministry of Health (MOH) reimbursement submission/approval, and infusion booking. Steps were fragmented, largely clinician-initiated, and patients frequently transported documents between services.

**Fig. 1:**
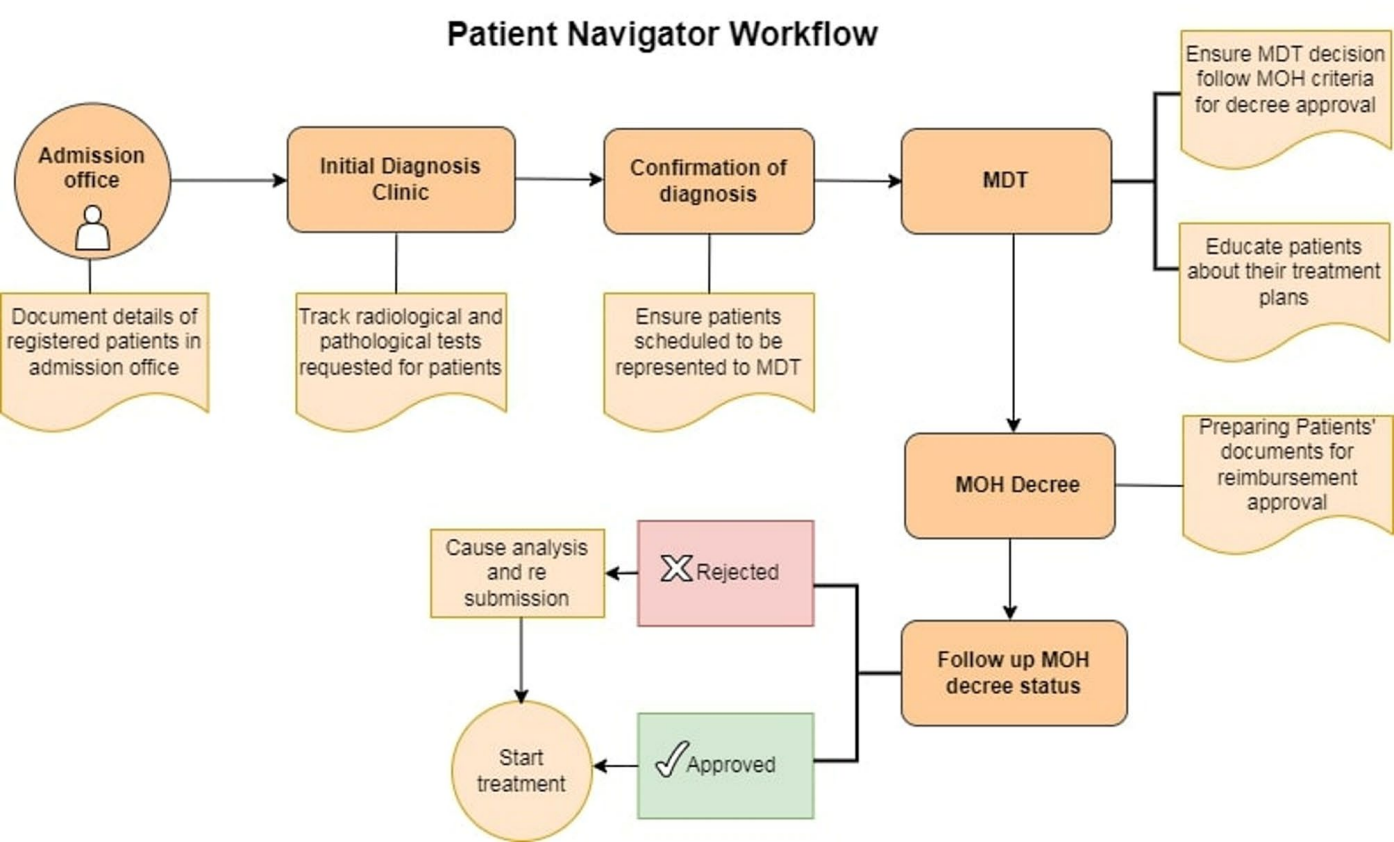
Patient Navigation Program workflow. Navigator-coordinated steps from admission through MDT, MOH approval, and treatment start. Activities include verifying MOH criteria at MDT, preparing/submitting reimbursement documents, tracking approval status, educating patients, and booking the earliest infusion slot upon approval. Abbreviations: MDT, multidisciplinary tumor board; MOH, Ministry of Health

**Post-implementation (with navigation).** The Patient Navigation Program (Fig. 1) deployed trained navigators who: (1) tracked completion of required diagnostics and ensured reports were available; (2) scheduled the earliest feasible MDT discussion; (3) assembled, prepared, and submitted complete MOH reimbursement files; (4) monitored MOH approval status and resolved deficiencies; and (5) booked the first available infusion appointment immediately upon approval.

### Study design

We conducted a retrospective cohort study at the Breast Cancer Comprehensive Center (BCCC) to evaluate the impact of the Patient Navigation Program on treatment timeliness for HER2-positive early breast cancer.

Patients were grouped as **without navigation** (before implementation) and **with navigation** (after implementation, with active navigator involvement). The program launched in mid-2022 and was fully operational from January 2023.

- **Primary analysis**: consecutive patients in the six months immediately before full implementation (July–December 2022; without navigation) versus the first six months after (January–June 2023; with navigation).
- **Sensitivity analysis**: the originally reported window of May–December 2022 (without navigation) versus January–December 2023 (with navigation) to assess robustness.

### Objectives

#### Primary objective

determine whether the Patient Navigation Program reduced the time from registration at BCCC to initiation of dual anti-HER2 therapy (start of trastuzumab/pertuzumab during neoadjuvant chemotherapy).

#### Secondary objectives

(1) decompose the total interval into component steps—registration→MDT decision, MDT decision→MOH submission, MOH submission→MOH approval, and MOH approval→therapy start—to localize improvements; (2) compare time from therapy initiation to surgery between cohorts; and (3) compare pathological complete response (pCR) rates between cohorts.

### Patient population

We included consecutive adults (≥ 18 years) with stage I–III HER2-positive invasive breast carcinoma (Immunohistochemistry (IHC)3 + or In situ hybridization (ISH)-amplified) planned for neoadjuvant chemotherapy plus dual anti-HER2 therapy during the study windows, who initiated neoadjuvant treatment at BCCC in 2022 or 2023.

The standard neoadjuvant protocol comprised an anthracycline–taxane backbone with dual HER2 blockade: four cycles of doxorubicin/cyclophosphamide (AC) followed by 12 weeks of weekly paclitaxel with concurrent trastuzumab and pertuzumab. Surgery is part of the standard pathway; however, surgery was not required for inclusion in time-interval analyses. Patients missing any key timestamp needed for interval computation were excluded from interval endpoints.

#### Exclusion criteria

de novo metastatic (stage IV) disease; no neoadjuvant dual HER2 blockade (e.g., contraindication to targeted therapy or upfront surgery/adjuvant approach).

### Data collection

**Fig. 2:**
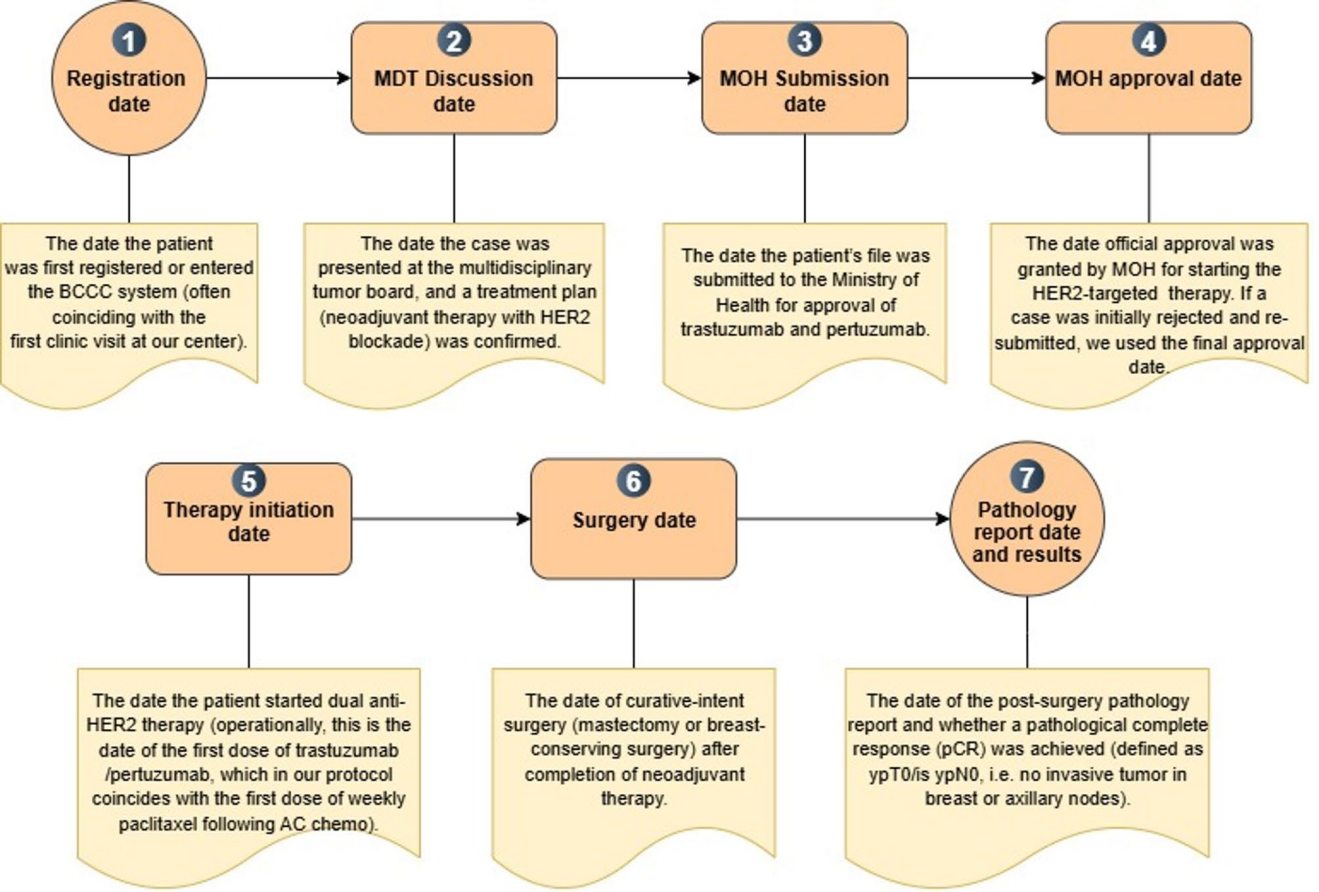
Timeline capture points. T1 (registration→MDT), T2 (MDT→MOH submission), T3 (submission→approval), T4 (approval→therapy start); plus, downstream dates (surgery; pathology/pCR). Abbreviations: MDT, multidisciplinary tumor board; MOH, Ministry of Health; pCR, pathological complete response; HER2, human epidermal growth factor 2; BCCC, breast comprehensive cancer center; AC, doxorubicin (Adriamycin) and cyclophosphamide

Data were abstracted retrospectively from the institutional electronic medical record (EMR) and patient files. Operational definitions and their positions along the pathway are mapped in Fig. 2, defining four prespecified intervals (T1–T4) and downstream dates (therapy initiation, surgery, pathology/pCR). All entries (expanded definition in supplementary Table 1 A & 1B) were double-checked; discrepancies or extreme outliers were adjudicated by chart review. We report observed intervals without inferring causality; occasional clinical reasons for delay (e.g., intercurrent illness, additional clearances, patient scheduling constraints) were documented during review.

### Endpoints

#### Primary endpoints

system time intervals—T1 (registration→MDT), T2 (MDT→MOH submission), T3 (MOH submission→MOH approval), T4 (MOH approval→therapy start), and their sum (**total time**).

#### Secondary endpoint

pCR among patients who underwent definitive surgery with evaluable pathology. Surgical procedures and outcomes were not prespecified primary endpoints.

### Statistical analysis

Baseline characteristics were summarized descriptively. For each interval (T1–T4, total), we compared **without-navigation (July–December 2022)** versus **with-navigation (January–June 2023)** using non-parametric mann-whitney u test; as normality wasn’t met. Where variances were unequal, Welch’s correction was used. Results are reported as mean (SD) or median (IQR) as appropriate, with mean differences and 95% confidence intervals. pCR rates were compared using chi-square tests. Statistical significance was set at *p* < 0.05 (two-sided). Sensitivity analyses repeated interval comparisons for **May–December 2022** versus **January–December 2023**. Analyses were conducted in **IBM SPSS Statistics v26**. Given the programmatic, retrospective design and similar year-to-year case mix, no multivariable adjustment was prespecified.

## Results

### Cohorts and analytic windows

The 2023 cohort (with navigation) represents the first full operational year of the Patient Navigation Program, coinciding with increasing referrals driven by national early-detection efforts. The 2022 cohort (without navigation) covered a partial year beginning in May. To improve comparability, our primary analysis was conducted using symmetric six-month windows: July–December 2022 (without navigation) versus January–June 2023 (with navigation). A sensitivity analysis was also performed comparing the entire available periods: May–December 2022 versus January–December 2023.

### Primary analysis: July–December 2022 vs. January–June 2023

#### Patient demographics and baseline

The two cohorts were demographically well balanced (Table 1). Mean age was 45.6 ± 10.3 years in 2022 and 46.4 ± 9.9 years in 2023. Referral patterns were similar, with most patients from Greater Cairo (78.3% vs. 70.0%), followed by Delta (10.0% vs. 16.7%), Upper Egypt (10.0% vs. 11.7%), and Suez/Ismailia/Red Sea (1.7% each).

**Table 1:** Baseline characteristics and pathological complete response (pCR) in the primary analysis cohorts (HER2-positive EBC treated with neoadjuvant chemotherapy + Dual anti her 2 therapy at BCCC)

Characteristic	2022 (Without navigation: Jul–Dec) *n* = 120	2023 (With navigation: Jan–Jun) *n* = 120	*p*-value
**Age**,** years** (mean ± SD)	45.6 ± 10.3	46.4 ± 9.9	—
**Geographic referral region**, n (column %)			
Greater Cairo	94 (78.3)	84 (70.0)	—
Delta	12 (10.0)	20 (16.7)	—
Upper Egypt	12 (10.0)	14 (11.7)	—
Suez/Ismailia/Red Sea	2 (1.7)	2 (1.7)	—
**Evaluable for pCR**, n	**115**	**117**	—
pCR (per pathology*), n (row %)			
Yes	78 (67.8)	81 (69.2)	**0.818**
No	37 (32.2)	36 (30.8)	

Abbreviations: BCCC, Breast Cancer Comprehensive Center; EBC, early breast cancer; pCR, pathological complete response

* pCR assessed among patients who underwent definitive surgery with evaluable pathology; percentages shown within the **evaluable** denominator for each cohort

Notes:

• Geographic rows sum to the cohort totals (***n*** **= 120** per column)

• pCR rows sum to the number **evaluable for pCR** (115 in 2022; 117 in 2023)

• “—” indicates not tested/not applicable for baseline descriptors

#### System intervals to therapy

The navigation program significantly shortened the MOH approval → therapy initiation interval (T4) (*p* = 0.008) (Table 2). Other intervals were not statistically different: registration → MDT (T1, *p* = 0.764), MDT → MOH submission (T2, *p* = 0.762), and submission → approval (T3, *p* = 0.678). The total time (registration → therapy) was modestly reduced but not statistically significant (*p* = 0.127).

**Table 2:** Between-cohort comparison of system time intervals (primary analysis: July–Dec 2022 vs. Jan–Jun 2023)

Interval	Median (Days) (Minimum – Maximum)	Test statistics (U)	*p*-value
**T1**: Registration → MDT	24 (0–360)	7.36	0.764
**T2**: MDT → MOH submission	54 (1–198)	6.34	0.762
**T3**: MOH submission → MOH approval	6 (0–15)	7.17	0.678
**T4**: MOH approval → therapy start	19 (0–168)	5.21	0.008
**Total**: Registration → therapy	124 (28–448)	6.38	0.127

Abbreviations: MDT, multidisciplinary tumor board; MOH, Ministry of Health

Notes: Comparisons are between without-navigation (July–December 2022) and with-navigation (January–June 2023) cohorts. U denotes the Mann–Whitney U test statistic as reported; p-values are two-sided. Lower interval values indicate faster progression to therapy

#### Pathological complete response (pCR)

pCR outcomes were similar: 78 patients in the 2022 cohort vs. 81 in 2023 achieved pCR (Table 1). The difference was not statistically significant (*p* = 0.818).

### Sensitivity analysis: May–December 2022 vs. January–December 2023

In a sensitivity analysis including 441 patients (162 in 2022; 279 in 2023), results were directionally consistent with the primary analysis and more robust: the total time from registration to initiation of dual anti-HER2 therapy decreased from 146.2 ± 76.6 days in 2022 to 121.6 ± 50.4 days in 2023 (difference − 24.6 days, *p* < 0.001). By interval, T1 (registration→MDT) improved from 44.4 to 36.6 days (*p* = 0.174), T2 (MDT→MOH submission) from 63.3 to 56.9 days (*p* = 0.108), T3 (submission→approval) increased slightly from 6.2 to 7.4 days (+ 1.2 days; *p* < 0.001), and T4 (approval→therapy start) shortened substantially from 32.4 to 20.8 days (–11.6 days; *p* < 0.001). pCR rates were similar (64.1% in 2022 vs. 69.9% in 2023; *p* = 0.76). Collectively, these data underscore that the program’s major effect was acceleration of T4, yielding a statistically significant reduction in overall time to therapy despite higher 2023 volumes (Table 3).

**Table 3:** Comparative time intervals from registration to dual HER2 therapy (Without- vs. With-Navigation)

Interval	2022 (Without-navigation) Mean ± SD (days)	2023 (With-navigation) Mean ± SD (days)	Mean Difference	*p*-value
T1: Registration → MDT	44.4 ± 69.0	36.6 ± 31.2	–7.8	0.174
T2: MDT → MOH Submission	63.3 ± 41.5	56.9 ± 34.3	–6.4	0.108
**T3**: Submission → Approval	6.2 ± 3.1	7.4 ± 2.90	+ 1.2	< 0.001
**T4**: Approval → Therapy	32.4 ± 28.1	20.8 ± 22.2	–11.6	< 0.001
**Total**: Registration → Therapy	146.2 ± 76.6	121.6 ± 50.4	–24.6	< 0.001
**pCR Rates**	**2022 (Without navigation)**	**2023 (With navigation)**		
	64.1%	69.9%		0.76

SD: standard deviation. Negative differences indicate faster times in 2023. Bolded intervals (T3, T4 and Total) had statistically significant reductions

## Discussion

Introducing a Patient Navigation Program at a high-volume public cancer center in an LMIC context was associated—on our primary, symmetric six-month analysis—with a targeted, statistically significant reduction in the MOH approval → therapy start interval (T4; *p* = 0.008) for patients with early HER2-positive breast cancer starting dual anti-HER2 therapy. Upstream intervals (registration→MDT, MDT→MOH submission, submission→approval) and the total registration→therapy time showed non-significant trends (*p* = 0.764, 0.762, 0.678, and 0.127, respectively), indicating that navigation most effectively addressed the operational bottleneck between approval and infusion scheduling—the segment most under institutional control. This pattern accords with global evidence that navigation mitigates system barriers and accelerates treatment initiation [9].

Our findings mirror experiences from multiple settings where navigation was adapted to local constraints yet consistently accelerated care. In the United States, navigation began as an equity-focused intervention in Harlem and targeted practical barriers such as insurance authorizations, transportation, appointment coordination, and communication, improving outcomes for underserved women [10]. Subsequent efforts—including the NCI-sponsored Patient Navigation Research Program—embedded navigators within clinic workflows to track each step from abnormal screening through diagnostic resolution to treatment initiation, with studies demonstrating faster resolution and higher odds of on-time treatment start [9, 10]. Taken together, the U.S. experience underscores that barrier-specific problem solving and proactive tracking—both core to our program—are key mechanisms of effect.

In Brazil, the national “60-day Law” requires public hospitals to start treatment within 60 days of diagnosis. Navigation teams in Sistema Único de Saúde (SUS) operationalized this mandate by monitoring the legal “clock,” assembling required documentation in advance, securing appointments across siloed services, and escalating delays to hospital leadership. Programs in Rio de Janeiro reported markedly shorter timelines and high compliance with the 60-day target when navigators were engaged [12]. As in Egypt, centralized coverage alone did not ensure timeliness; navigation provided the coordination layer that translated funding into punctual care.

In Rwanda, where workforce and geographic constraints are pronounced, a digitally enabled multi-hospital navigation model linked peripheral and referral centers, standardized referral forms, flagged missing diagnostics, and used dashboards to shorten handoffs across pathology, imaging, and oncology clinics. Reported time from diagnosis to treatment start fell substantially after implementation [14]. This experience highlights the additive value of digital tracking alongside human navigators—a direction that could further compress intervals within Egypt’s largely paper-based approval environment.

Across these contexts, navigation is flexible and transferable: it targets the dominant barriers in each system—socioeconomic/insurance (U.S.), legal timeframes and service fragmentation (Brazil), workforce and geography (Rwanda), or administrative bottlenecks and rising volumes (Egypt)—yet converges on the same outcome: faster movement to definitive therapy [9, 10, 12, 14].

Our sensitivity analysis reinforced these conclusions: when all available months were included (May–December 2022 vs. January–December 2023), the total time from registration to dual anti-HER2 therapy decreased by 24.5 days (*p* < 0.001), driven largely by a substantial improvement in T4 (–11.6 days; *p* < 0.001), with a small increase in T3 likely reflecting external payer processing dynamics under higher 2023 volumes. Together, the primary analysis pinpoints where navigation works (post-approval operations), and the sensitivity analysis shows that, over a full operational year with increasing referrals, these gains translate into a clinically meaningful reduction in overall time to therapy.

Although pCR rates were similar across cohorts, the slight favorable direction is clinically relevant given the strong association between pCR and improved long-term outcomes in HER2-positive disease [8, 18]. By reducing delays, navigation may lessen pre-treatment tumor growth and enhance chemosensitivity—mechanisms consistent with international evidence linking prompt care to better outcomes [9]. Navigation’s additional benefits—improved knowledge, communication, and adherence—are well documented and were reflected in our early implementation experience [9, 16].

These results align with the WHO Global Breast Cancer Initiative (GBCI) emphasis on timely diagnosis, streamlined referral pathways, and coordinated treatment in resource-constrained settings [15]. Embedding navigation within national cancer control plans, while digitizing tracking and inter-facility referrals, could further compress upstream intervals (T1–T2) and stabilize payer-facing timelines (T3) [13, 14, 17]. In publicly funded systems, navigation provides the operational glue that turns policy and coverage into timely, equitable access to evidence-based therapy.

In summary, the BCCC Patient Navigation Program produced a precise, measurable improvement in the most actionable segment of the pathway (T4) in the primary analysis and a significant reduction in total time to therapy over the full operational year. Consistent with international experience, navigation is a scalable, equity-promoting strategy for LMIC cancer centers progressing toward GBCI targets [9, 10, 12–15, 17].

## Limitations

Our study has several limitations consistent with a before–after, retrospective design in health services research. First, without randomization or a concurrent control group, causal attribution to the Patient Navigation Program cannot be assured. Although we prespecified symmetric six-month windows for the primary analysis (July–December 2022 vs. January–June 2023) to improve comparability and used a full-year sensitivity analysis, secular trends and unmeasured confounding may persist (e.g., rising referrals from national early-detection activities, informal workflow refinements, or learning-curve effects).

Second, this is a single-center study from a large tertiary public cancer hospital in Egypt; the organizational context (e.g., staffing, MOH approval pathways, infusion capacity) may limit generalizability to smaller facilities or different provinces. Third, interval measurement relied on EMR timestamps and paper approvals. Despite double-checking and adjudication, residual measurement error is possible; exclusions for missing key dates may introduce selection bias.

Fourth, the study was not powered to detect modest differences in pCR and did not include time-to-event outcomes (recurrence, survival), patient-reported outcomes, or cost-effectiveness. pCR was assessed only among surgical cases with evaluable pathology, which may introduce attrition bias. Finally, we did not apply multivariable adjustment or quasi-experimental methods (e.g., interrupted time series, difference-in-differences) that could further address confounding by time-varying factors, detailed navigator metrics (staff numbers, caseloads, training hours, costs) were not prospectively collected.

Future work should include multi-center prospective designs, equity-focused subgroup analyses, Patient -Reported Outcomes (PROs), survival endpoints, cost-effectiveness, and implementation outcomes (acceptability, fidelity, reach).

## Conclusion

In conclusion, the implementation of a Patient Navigation Program at our tertiary cancer center in Egypt significantly reduced treatment delays for HER2-positive early breast cancer, particularly by shortening the critical interval between MOH approval and initiation of dual anti-HER2 therapy. When examined across the full operational year, navigation achieved a meaningful reduction in overall time to treatment despite rising referral volumes, underscoring its capacity to deliver system-level efficiency gains in resource-constrained settings. While pCR rates were comparable, the timeliness gains are clinically important given the established association between prompt therapy and improved outcomes. These findings demonstrate that navigation is a practical, adaptable, and equity-promoting intervention aligned with global initiatives to strengthen cancer care in LMICs, and they provide a compelling rationale for continued implementation, scale-up, and prospective evaluation across similar health systems.

## Supplementary Information

Below is the link to the electronic supplementary material.

Supplementary Material 1

## Data Availability

The datasets generated and analyzed during the current study are not publicly available due to hospital privacy regulations but are available from the corresponding author on reasonable request (with appropriate institutional approvals).

## Abbreviations

AC: Doxorubicin (Adriamycin) and cyclophosphamide
BCCC: Breast Cancer Comprehensive Center
BHGI: Breast Health Global Initiative
EBC: Early breast cancer
EMR: Electronic medical record
ER: Estrogen receptor
HER2: Human epidermal growth factor receptor 2
HR: Hormone receptor
IHC: Immunohistochemistry
IRB: Institutional Review Board
ISH: In situ hybridization
LMIC: Low- and middle-income country
MDT: Multidisciplinary tumor board
MOH: Ministry of Health
pCR: Pathological complete response
PROs: Patient-reported Outcomes
SD: Standard deviation
SUS: Sistema Único de Saúde
T1–T4: Prespecified time intervals (T1: registration →MDT; T2: MDT →MOH submission; T3: MOH submission →MOH approval; T4: MOH approval →therapy initiation)
WHO: World Health Organization
