# Supplementary material for: Streamlining care through patient navigation: a retrospective cohort study of timely anti-HER2 therapy in early breast cancer in a low-middle income country
Source: BMC Health Serv Res. 2025 Nov 8;25:1454. doi: 10.1186/s12913-025-13606-8 (PMC12595620; doi:10.1186/s12913-025-13606-8)
Supplement: Supplementary file 1 — Supplementary Material 1 [file 12913_2025_13606_MOESM1_ESM.docx]

**Supplementary Table 1. Timeline data elements and derived intervals**

**A. Raw date fields (capture from EMR/patient file)**

| **Field (short name)** | **Operational definition** | **Source** | **Format** |
| --- | --- | --- | --- |
| Registration date (Registration_Date) | Date the patient first registered/entered the BCCC system (often the first clinic visit). | EMR + patient file | YYYY-MM-DD |
| MDT discussion date (MDT_Discussion_Date) | Date the case was presented at the multidisciplinary tumor board and neoadjuvant HER2 blockade plan confirmed. | EMR + MDT minutes | YYYY-MM-DD |
| MOH submission date (MOH_Submission_Date) | Date the file was submitted to the Ministry of Health for trastuzumab + pertuzumab approval. | EMR + reimbursement file | YYYY-MM-DD |
| MOH approval date (MOH_Approval_Date) | Date official MOH approval was granted to start HER2-targeted therapy; if re-submitted, use final approval date. | EMR + reimbursement file | YYYY-MM-DD |
| Therapy initiation date (Therapy_Initiation_Date) | Date of the **first dose of trastuzumab/pertuzumab** (in our protocol, coincides with first weekly paclitaxel after AC). | EMR + chemo orders | YYYY-MM-DD |
| Surgery date (Surgery_Date) | Date of curative-intent surgery (mastectomy or breast-conserving surgery) post-neoadjuvant therapy. | EMR + operative note | YYYY-MM-DD |
| Pathology report date (Pathology_Report_Date) | Date of final post-surgery pathology report. | EMR + pathology report | YYYY-MM-DD |
| pCR status (pCR_Status) | Pathologic complete response achieved (ypT0/is ypN0: no invasive tumor in breast or axillary nodes). | Pathology report | Yes/No |

**B. Derived time intervals (days)**

| **Interval** | **Name** | **Definition** | **Computation** |
| --- | --- | --- | --- |
| T1 | Registration → MDT | Time from Registration to MDT discussion | MDT_Discussion_Date − Registration_Date |
| T2 | MDT → MOH submission | Time from MDT discussion to MOH submission | MOH_Submission_Date − MDT_Discussion_Date |
| T3 | Submission → MOH approval | Time from MOH submission to MOH approval decision | MOH_Approval_Date − MOH_Submission_Date |
| T4 | MOH approval → Therapy | Time from MOH approval to first HER2 therapy dose | Therapy_Initiation_Date − MOH_Approval_Date |
| Total time | Registration → Therapy | Overall time from Registration to Therapy initiation | Therapy_Initiation_Date − Registration_Date (≈ T1+T2+T3+T4 when complete) |
| Therapy → Surgery | Therapy to Surgery | Time from first HER2 therapy dose to Surgery | Surgery_Date − Therapy_Initiation_Date |

*Abbreviations:* EMR, Electronic Medical Record; BCCC, Breast Cancer Comprehensive Center; MDT, multidisciplinary team; MOH, Ministry of Health; HER2, Human Epidermal growth factor Receptor 2; AC, Doxorubicin (Adriamycin) and cyclophosphamide; pCR, pathological complete response;
